# Smartphone Addiction and Its Psychosocial Correlates Among University Students

**DOI:** 10.7759/cureus.91165

**Published:** 2025-08-28

**Authors:** Mohammed A Alhassan, Emad M Al-Otaibi, Meshal S Bin Ofaysan, Khalid H Alarjani, Maan O Alzuhairi, Nawaf S Alarfaj, Faisal S Al-murayt, Bader M Alotaibi, Khalid F Alanazi, Abdulelah M Alfawaz, Anas Musa

**Affiliations:** 1 Pediatric Medicine, Prince Sattam Bin Abdulaziz University, Al-kharj, SAU; 2 Public Health, Prince Sattam Bin Abdulaziz University, Al-kharj, SAU; 3 Cardiology, Rashid Hospital, Abu Dhabi, ARE

**Keywords:** anxiety, depression, exercise, internet addiction disorder, loneliness, mental health, sleep quality

## Abstract

Background: Addiction to smartphones is an increasingly recognized behavioral issue among university students. It has potentially adverse effects on mental and psychosocial well-being.

Objectives: To assess the prevalence of smartphone addiction and examine its associations with six psychosocial and behavioral health measures among university students.

Methods: A cross-sectional, web-based survey was conducted among university students in Saudi Arabia. Using convenience sampling, we collected demographic data along with validated instruments: the 10-item Smartphone Addiction Scale-Short Version (SAS-SV) for addiction risk, and five additional validated scales for assessing depression, anxiety, sleep quality, physical activity, and social connectedness. Data were analyzed using Stata 17 (StatCorp LLC, College Station, TX) with descriptive statistics, between-group tests (t‑tests, Mann-Whitney U, χ²), Spearman correlations, and crude logistic regression analyses.

Results: A total of 383 students completed the survey, with a mean daily smartphone use of 7.6 ± 3.5 hrs. Overall, 268 (70.0%) individuals screened at risk for smartphone addiction, with no significant differences by gender, age, or academic grades. However, a higher risk was observed in computer engineering and business students (p = 0.043) and among those who used phones for longer periods daily (p < 0.001). Probable depression and anxiety were present in 176 (45.9%) and 162 (42.3%) participants, respectively, and were associated with increased odds of smartphone addiction (odds ratio (OR) = 3.83, 95 % CI 2.35-6.25; OR = 3.11, 95 % CI 1.91-5.06). Physical activity and better sleep quality were inversely associated with addiction risk (OR = 0.54, 95 % CI 0.34-0.86; OR = 0.78, 95 % CI 0.63-0.96), while loneliness (34.0% vs. 15.7%, p < 0.001) and negative perceptions of smartphone impact (OR = 3.50, p = 0.001) correlated with higher addiction risk. Smartphone‑addiction scores correlated positively with depression (r = 0.43) and anxiety (r = 0.35) and negatively with sleep quality (r = -0.13) and physical activity (r = -0.20) scores (all p < 0.05). Frequent engagement in creative activities (OR = 0.47, p = 0.018) was associated with reduced odds of smartphone addiction.

Conclusion: Smartphone addiction risk was exceptionally high and strongly linked to depression, anxiety, and loneliness, whereas better sleep, regular physical activity, and creative engagement were protective. These findings reiterate the need for integrated interventions targeting students, combining digital literacy education, mental‑health screening, and lifestyle promotion.

## Introduction

Smartphones have become an integral part of the daily lives of young adults, particularly university students, whose academic, social, and recreational activities often revolve around digital platforms. Although these devices offer significant benefits, excessive or maladaptive use has raised concerns regarding their impact on mental health, sleep, academic performance, and general well-being [1]. The concept of smartphone addiction, sometimes referred to as problematic smartphone use, has gained increasing recognition as a behavioral addiction characterized by compulsive usage, withdrawal symptoms, and functional impairments [2].

Prevalence estimates vary widely across settings, with studies reporting smartphone addiction risks affecting 20% to over 60% of university students globally [3]. Such overuse has been associated with depression, anxiety, poor sleep quality, physical inactivity, and decreased academic functioning [4]. In Saudi Arabia, where smartphone penetration is among the highest in the world [5], the university student population may be particularly vulnerable to the adverse effects of digital overuse.

Despite growing concern about the mental health and behavioral consequences of excessive smartphone use, only a few studies have examined the interplay between smartphone addiction and psychosocial and behavioral correlations in this population [6,7]. To our knowledge, no study in Saudi Arabia has yet examined how depression, anxiety, sleep quality, physical activity, social connectedness, and engagement in creative activities interact in this context among university students. This study seeks to fill these gaps by estimating current smartphone addiction prevalence and exploring its associations with six key psychosocial and behavioral measures: depression, anxiety, sleep quality, physical activity, social connectedness, and engagement in creative activities. We included the frequency of engagement in creative activities as an exploratory behavioral correlate to examine its association with smartphone addiction risk.

## Materials and methods

Study design and setting

We conducted a web-based survey among students at Prince Sattam Bin Abdulaziz University in Saudi Arabia from August 2024 to April 2025. The university includes various colleges across medical, engineering, business, and humanities disciplines.

Inclusions and sampling

Full-time undergraduate students were eligible for inclusion. Students who were part-time, had withdrawn from the semester, or were exchange students were excluded from the study. A non-probability convenience sampling strategy was used. Data collectors identified WhatsApp groups (end-to-end encrypted) representing various student batches across multiple colleges without applying predefined selection criteria. The web-based questionnaire link was distributed to these groups, and all members were invited to participate voluntarily. Only responses submitted through the online form were included in the analysis. A minimum sample size of 293 was estimated for a 95% confidence level, 5% margin of error, design effect of 1, population size of ~26,000, and hypothesized prevalence of 26% [8]; the achieved sample was 383.

Ethical considerations

The study was approved by the Standing Committee of Bioethics Research of Prince Sattam Bin Abdulaziz University (SCBR-441/2025). Informed consent was obtained electronically via a checkbox prior to survey initiation. All data were collected anonymously and treated confidentially.

Information obtained and measures

Sociodemographic data, Grade Point Average, hours of smartphone use per day, primary purpose of smartphone use, and perceived effect on academic performance were obtained. Overall perceived impact of smartphone use was captured with a single item (positive,’ ‘negative,’ ‘neutral’). The frequency of engagement in creative activities was assessed through a structured questionnaire: “How often do you engage in creative or hands-on activities (e.g., drawing, crafting, playing a musical instrument, cooking) in your free time?” with three Likert options. Validated measures were used to assess Smartphone addiction using the 10-item Smartphone Addiction Scale-Short Version (SAS-SV), scored on a 6-point Likert scale. Gender-specific cut-offs (males ≥31, females ≥33) were used to define addiction risk [9].

Depression and anxiety were assessed using the Patient Health Questionnaire-2 (PHQ-2) [10] and Generalized Anxiety Disorder-2 (GAD-2) [11] instruments, respectively, with a score ≥3 indicating probable major depression or anxiety disorder. PHQ-2 and GAD-2 are screening instruments indicating probable symptoms and do not constitute clinical diagnoses. Sleep quality was measured using the Single-Item Sleep Quality Scale (SQS), a validated 0-10 visual analogue scale, categorized into five levels from terrible to excellent [12]. Physical activity was evaluated using the Exercise Vital Sign (EVS) [13], categorizing participants as physically active (≥150 minutes/week) or inactive (<150 minutes/week). Social connectedness was assessed via a validated 3-item scale, scored from 0 to 9, with higher scores indicating greater perceived connectedness [14]. The questionnaire was prepared in English and Modern Standard Arabic [15] and pilot-tested among 10 students to assess clarity, formatting, and flow; no issues were identified, and no changes were required. All instruments are publicly available for academic use and require no formal permission beyond proper citation; we used the original versions without modification.

Data handling and statistical analysis

Data were analyzed using Stata version 17, 2021 (StataCorp LLC, College Station, TX). Visual inspection of histograms and Q-Q plots was used to assess normality. Descriptive statistics were reported as means (SD) or frequencies (%). Between-group comparisons used t-tests, Mann-Whitney U tests, and chi-square tests as appropriate. Correlation analyses were conducted using Spearman’s rho. Crude odds ratios (OR) were calculated using simple logistic regression. Because no a priori confounder set was specified and the analysis was exploratory, adjusted multivariable models were not fitted, and associations were interpreted descriptively. Figures were generated with assistance from Julius AI (Infographics Generation AI chatbot, Caesar Labs, Inc., 2025, July 21) and verified in Stata.

## Results

Participant characteristics and smartphone addiction

A total of 383 university students participated in the study. Most participants were enrolled in Medicine (20.9%; n=80), Humanities (18.8%; n=72), and Computer Engineering (16.7%; n=64). The majority (80.2%; n=307) of students lived with their families. The primary reported purposes for smartphone use included social media (30.8%; n=118) and watching videos (25.6%; n=98), while academic purposes constituted 15.1% (n=58). The mean daily smartphone usage was 7.64 hours (SD = 3.46) (Table 1).

**Table 1:** Characteristics of participants by smartphone addiction risk (N = 383) * Represents a significant p-value. Categorical comparisons used χ² tests; continuous/ordinal variables used t-tests or Mann–Whitney U tests as appropriate. Cells are n (row %); the Total column is n (% of N=383).

Variable	Category	Not at Risk; n = 115 (30.0%)	At Risk; n = 268 (70.0%)	Total; n = 383	p-value
Gender	Male	75 (28.2%)	191 (71.8%)	266 (69.5%)	0.239
	Female	40 (34.2%)	77 (65.8%)	117 (30.5%)	
Age (years; median (IQR))	–	21 (20, 22)	21 (20, 22)	21 (20, 22)	0.115
Marital Status	Single	110 (29.4%)	264 (70.6%)	374 (97.7%)	0.125
	Married	2 (40.0%)	3 (60.0%)	5 (1.3%)	
	Divorced/Widowed	3 (75.0%)	1 (25.0%)	4 (1.0%)	
College	Medicine	20 (25.0%)	60 (75.0%)	80 (20.9%)	0.043*
	Law	8 (42.1%)	11 (57.9%)	19 (5.0%)	
	Applied Med Sciences	11 (35.5%)	20 (64.5%)	31 (8.1%)	
	Pharmacy	6 (24.0%)	19 (76.0%)	25 (6.5%)	
	Computer Engineering	12 (18.8%)	52 (81.3%)	64 (16.7%)	
	Engineering	8 (34.8%)	15 (65.2%)	23 (6.0%)	
	Business Administration	8 (21.1%)	30 (78.9%)	38 (9.9%)	
	Humanities	32 (44.4%)	40 (55.6%)	72 (18.8%)	
	Other	10 (32.3%)	21 (67.7%)	31 (8.1%)	
Academic Year	–	–	–	–	0.519
Accommodation	With Family	97 (31.6%)	210 (68.4%)	307 (80.2%)	0.178
	Dormitory	18 (23.7%)	58 (76.3%)	76 (19.8%)	
Smartphone Use (hrs/day); mean (SD)	–	6.5 (3.3)	8.2 (3.4)	7.6 (3.5)	<0.001*
Primary Use Purpose	Academic	22 (37.9%)	36 (62.1%)	58 (15.1%)	0.532
	General reading	12 (41.4%)	17 (58.6%)	29 (7.6%)	
	Gaming	6 (31.6%)	13 (68.4%)	19 (5.0%)	
	Messaging/communication	11 (24.4%)	34 (75.6%)	45 (11.7%)	
	Watching videos	28 (28.6%)	70 (71.4%)	98 (25.6%)	
	Social media	31 (26.3%)	87 (73.7%)	118 (30.8%)	
	Other	5 (31.3%)	11 (68.8%)	16 (4.2%)	
GPA; median (IQR)	–	4 (3.5, 4.5)	4 (3.5, 4.5)	4 (3.5, 4.5)	0.395

The mean score on the SAS-SV among participants was 37.48 (SD = 10.47). Based on established cutoffs, 268 students (70.0%) were classified as being at risk of smartphone addiction. Out of the total male respondents, 191 (71.80%) were found to be at risk of smartphone addiction, while 77 (65.81%) of the total female respondents were at risk. There was no statistically significant association between smartphone addiction risk and gender (p = 0.239), age (p = 0.115), or GPA (p = 0.395). A significant association was observed between academic discipline and addiction risk (p = 0.043). Higher proportions of students at risk of smartphone addiction were found in the Colleges of Computer Engineering and Business Administration, while the proportion was lower among students in Humanities. Students at risk of addiction reported longer daily smartphone usage (p < 0.001). No significant differences were found between groups regarding the primary purpose of smartphone use (p = 0.532) (Table 1).

Associations with mental health and lifestyle variables

Probable major depression (PHQ-2 ≥ 3) and potential anxiety (GAD-2 ≥ 3) were found in 45.9% (n=176) and 42.3% (n=162) of students, respectively. Poor/terrible sleep quality (as measured by SQS) and physical inactivity (as measured by Exercise Vital Sign (EVS)) were reported by 30.5% (n = 117) and 69.5% (n = 266) of students, respectively (Table 2).

**Table 2:** Descriptive statistics for phone addiction, psychological, sleep, and physical activity measures (N = 383) EVS: Exercise Vital Sign.

Variable	Category / Metric	n	% / Value
Smartphone Addiction (SAS-SV)	Mean (SD)	—	37.48 (10.47)
	At risk of smartphone addiction	268	70.0%
	Not at risk	115	30.0%
Depression (PHQ-2)	Probable major depression	176	45.9%
	Not likely depressed	207	54.1%
Anxiety (GAD-2)	Probable anxiety disorder	162	42.3%
	Not likely anxious	221	57.7%
Sleep Quality (SQS)	Terrible	33	8.6%
	Poor	84	21.9%
	Fair	157	41.0%
	Good	77	20.1%
	Excellent	32	8.4%
Physical Activity (EVS)	Physically inactive	266	69.5%
	Physically active	117	30.5%

**Figure 1:**
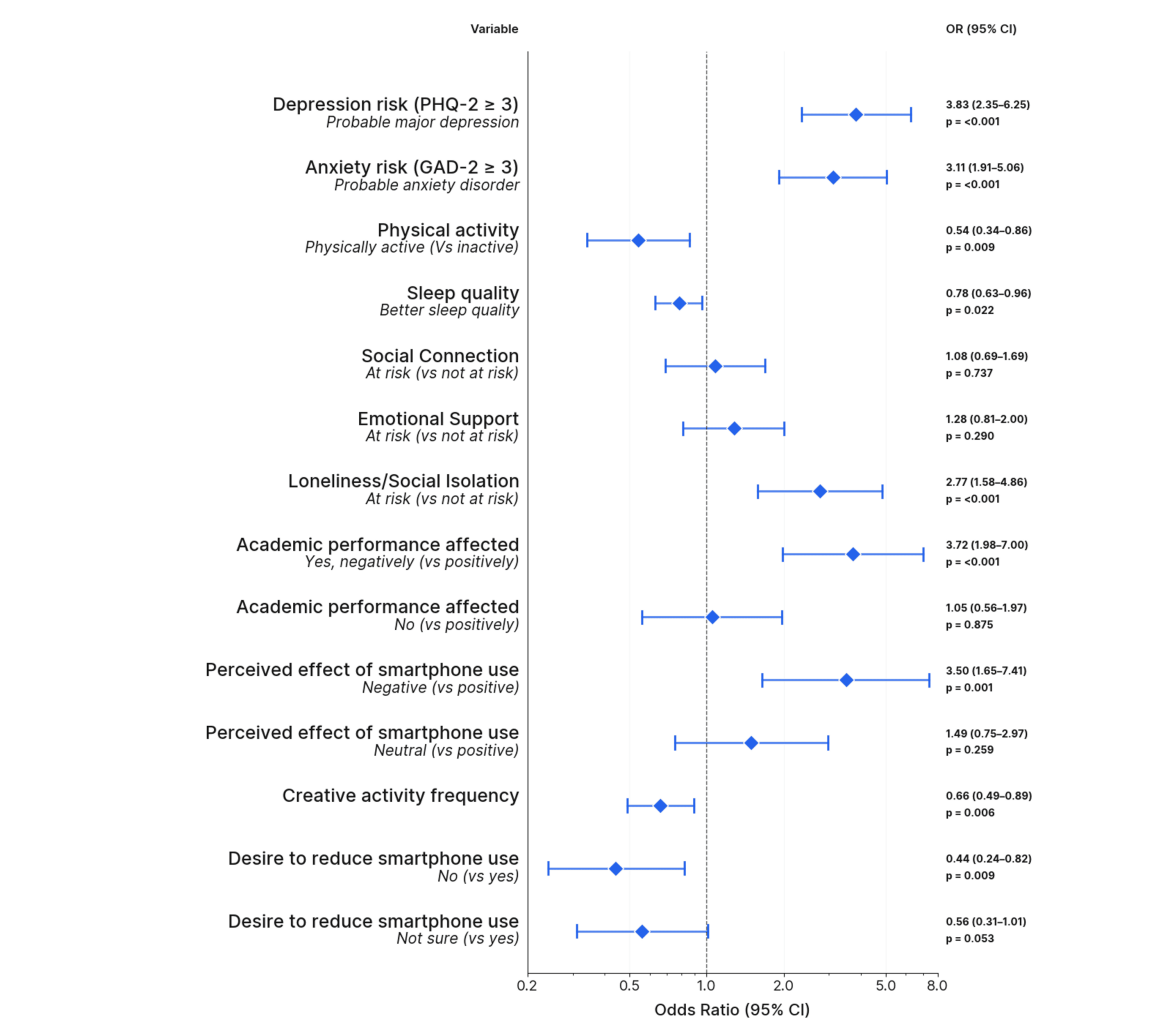
Univariate associations between selected variables and risk of smartphone addiction among university students (N = 383) Effect on academic performance is perceived, not actual grades.

Crude odds of smartphone addiction were significantly higher in students with probable depression (OR = 3.83, 95% CI: 2.35-6.25) and anxiety (OR = 3.11, 95% CI: 1.91-5.06). Being physically active was associated with significantly lower odds of smartphone addiction (OR = 0.54; 95% CI: 0.34-0.86; p = 0.009). Sleep quality was inversely associated with addiction risk (OR = 0.78; 95% CI: 0.63-0.96; p = 0.022). A significant association was found between loneliness/social isolation and smartphone addiction risk (p < 0.001). Participants reporting frequent feelings of loneliness or isolation were substantially more likely to exhibit signs of smartphone addiction (34.0% vs. 15.7%; p< 0.001). In contrast, there was no significant association between social connection frequency or emotional support and smartphone addiction risk (p = 0.737 and p = 0.289, respectively). Frequent engagement in creative activities (OR = 0.47, p = 0.018) was associated with reduced odds of smartphone addiction. Negative perceptions of smartphone impact were linked to higher addiction risk (OR = 3.50, p = 0.001) (Figure 1 and appendix).

**Figure 2:**
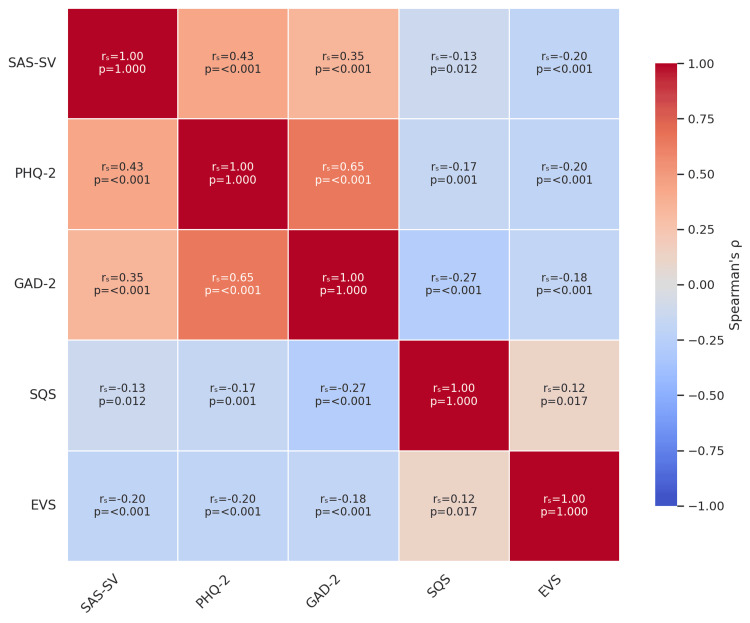
Correlation matrix of smartphone addiction and psychosocial/behavioral variables Spearman’s rank correlation coefficients (rₛ) and corresponding p-values are displayed for the associations between smartphone addiction (SAS-SV), depressive symptoms (PHQ-2), anxiety symptoms (GAD-2), sleep quality (SQS), and physical activity (EVS). Positive values (red hues) indicate direct associations, while negative values (blue hues) indicate inverse associations.

**Figure 3:**
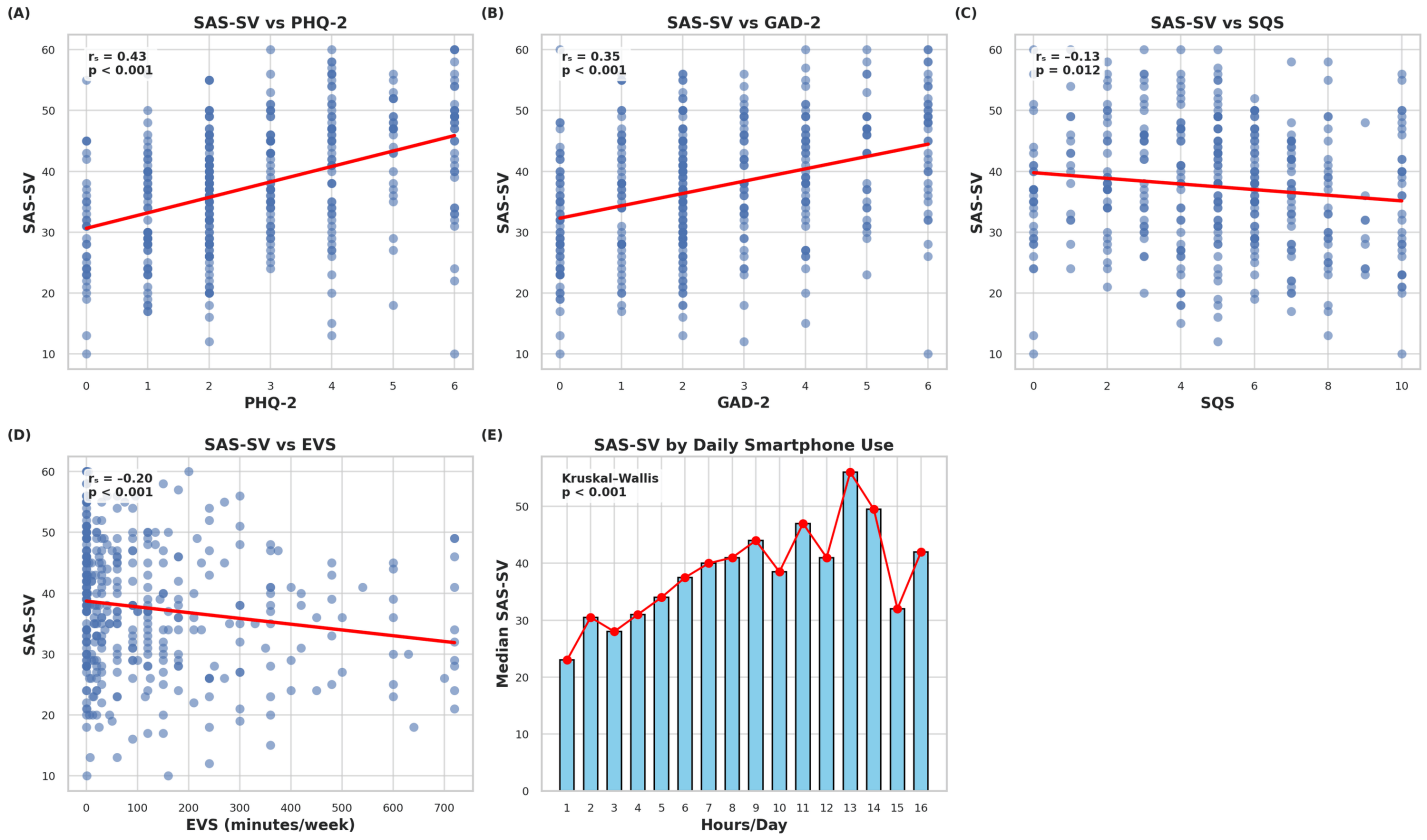
Correlations between smartphone addiction and psychosocial/behavioral variables Four plots illustrate the relationships between smartphone addiction (SAS-SV scores) and depressive symptoms (PHQ-2) (A), anxiety symptoms (GAD-2) (B), sleep quality (SQS) (C), and physical activity levels (EVS; minutes/week) (D). A bar plot shows median SAS-SV scores by rounded hours of daily smartphone use (E).

Smartphone addiction scores were significantly correlated with probable major depression (r = 0.43, p < .001), generalized anxiety (r = 0.35, p < .001), and negatively with sleep quality (r = -0.13, p = .012) and physical activity (r = -0.20, p < .001). PHQ-2 and GAD-2 scores were strongly positively correlated and both showed inverse associations with SQS and EVS (Figures 2, 3).

## Discussion

**Figure 4:**
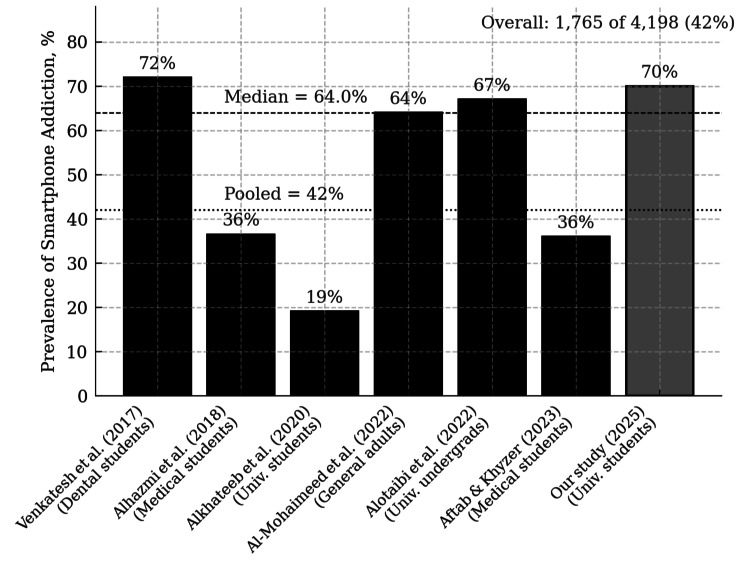
Prevalence of smartphone addiction in Saudi‐based studies that used the Smartphone Addiction Scales (SAS or SAS‑SV), 2017–2025 Image Credit: Mohammed Abdulrahman Alhassan References [7, 16-20].

This study found a high prevalence of smartphone addiction risk among university students in Saudi Arabia, consistent with prior regional studies. Across seven Saudi investigations that employed the Smartphone Addiction Scale or its short version between 2017 and 2025, a total of 4,147 participants were surveyed, and 1,731 (42 %) met addiction criteria (Figure 4). Prevalence ranged from 19.1 % (371 of 1,941) in a multicenter university sample [16] to 71.9 % (136 of 189) among dental students [17]. A community adult survey found 64.0 % (453 of 708) [7], while medical students' estimates were 36.5 % (66 of 181) [18] and 36.0 % (72 of 200) [19]. Another undergraduate cohort reported 67.0 % (365 of 545) [20], and our present study recorded 70.0 % (268 of 383) (Figure 4). With the exception of the 2020 university survey, all estimates exceed the recent global pooled prevalence of smartphone addiction (26.99 %; 95 % CI 22.73-31.73) reported in a 64-country meta-analysis [8].

Given the consistently high risk observed, particularly among health science undergraduates, Saudi universities and public health agencies should consider targeted screening and digital‑literacy interventions. We identified a greater number of students willing to reduce their time spent using smartphones, a point that might encourage preventive interventions. Future research ought to adopt nationally representative sampling frames and employ prospective designs to elucidate longitudinal health impacts and modifiable risk factors.

Excessive smartphone use was associated with depressive and anxiety symptoms; students with probable depression or anxiety had significantly higher odds of smartphone addiction, more than threefold in both cases. These findings are in line with prior studies that highlight maladaptive smartphone use as both a consequence and a contributor to psychological distress [21]. Engagement in protective health behaviors, such as regular physical activity and better sleep, was inversely associated with addiction risk. These findings support theories of behavioral displacement, where excessive smartphone use may erode time and motivation for sleep and exercise [22]. Engagement in creative or hands-on activities was associated with lower odds of smartphone addiction; these activities may warrant evaluation as potential strategies in future research. Even occasional involvement in creative or hands-on activities (e.g., arts, writing, manual tasks) was associated with significantly reduced odds of addiction. This pattern is consistent with the possibility that alternative rewarding activities are associated with a lower likelihood of compulsive smartphone behaviors [23].

Perceptions about smartphone use were also strongly associated with addiction risk. Students who viewed smartphone use as academically detrimental or overall negative were significantly more likely to be classified as addicted. These findings may reflect retrospective awareness of negative impacts or internal conflict associated with use patterns. Only loneliness showed a significant relationship with smartphone addiction, consistent with prior studies linking subjective social isolation to problematic technology use [24]. In contrast, structural and perceived support domains were not predictive, highlighting the unique role of felt loneliness in digital overdependence.

We believe the strength of our study lies in being, to our knowledge, the first in Saudi Arabia to investigate the interrelationships among six psychosocial constructs simultaneously using validated instruments. However, the cross-sectional design limits causal inference, and convenience sampling without a known response rate may affect generalizability. As participation was based on accessibility and willingness to respond, the sample may not be fully representative of the broader university student population, and findings should be interpreted with consideration of potential selection bias. Data were collected from August 2024 to April 2025, which may introduce temporal heterogeneity. All measures were self-reported and may be subject to recall and social desirability bias. Reported odds ratios are crude (unadjusted) and should be interpreted as exploratory associations.

## Conclusions

In conclusion, smartphone addiction risk affected 70% of our undergraduates and 42% of 4,198 Saudi participants pooled from seven SAS/SAS‑SV studies, confirming a substantial national burden. Depressive and anxiety symptoms were associated with roughly threefold higher odds of smartphone addiction risk, whereas better sleep quality, regular physical activity, and engagement in creative activities were associated with lower odds; students reporting loneliness also had higher odds of addiction. These findings highlight the complex interplay between psychological and behavioral factors that drive excessive smartphone use. University health programs may consider pairing digital-literacy training with mental health screening and lifestyle promotion initiatives. Future representative longitudinal studies are needed to clarify causal pathways and test multifaceted interventions.

## Appendices

**Table 3:** Univariate associations with risk of smartphone addiction (N = 383) * Represents a significant p-value.

Variable	Category	Not at risk, n (%)	At risk, n (%)	Total (N)	χ² / z	p-value
Depression risk (PHQ-2 ≥ 3)	Not likely depressed	87 (42.0%)	120 (58.0%)	207	χ² = 29.66	<0.001*
	Probable major depression	28 (15.9%)	148 (84.1%)	176		
Anxiety risk (GAD-2 ≥ 3)	Not likely anxious	87 (39.4%)	134 (60.6%)	221	χ² = 20.66	<0.001*
	Probable anxiety disorder	28 (17.3%)	134 (82.7%)	162		
Sleep quality	Terrible	11 (33.3%)	22 (66.7%)	33	χ² = 10.14	0.038*
	Poor	16 (19.1%)	68 (81.0%)	84		
	Fair	45 (28.7%)	112 (71.3%)	157		
	Good	29 (37.7%)	48 (62.3%)	77		
	Excellent	14 (43.8%)	18 (56.2%)	32		
Physical activity	Physically inactive	69 (25.9%)	197 (74.1%)	266	χ² = 6.30	0.012*
	Physically active	46 (39.3%)	71 (60.7%)	117		
Social Connection	Not at risk	45 (39.1%)	100 (37.3%)	145 (37.9%)	χ² = 0.11	0.737
	At risk of social disconnection	70 (60.9%)	168 (62.7%)	238 (62.1%)		
Emotional Support	Not at risk	46 (40.0%)	92 (34.3%)	138 (36.0%)	χ² = 1.12	0.289
	At risk of lacking emotional support	69 (60.0%)	176 (65.7%)	245 (64.0%)		
Loneliness/Social Isolation	Not at risk	97 (84.4%)	177 (66.0%)	274 (71.5%)	χ² = 13.24	<0.001*
	At risk	18 (15.7%)	91 (34.0%)	109 (28.5%)		
Smartphone use affected academics?	Yes, positively	51 (35.2%)	94 (64.8%)	145	χ² = 30.24	<0.001*
	Yes, negatively	16 (12.5%)	112 (87.5%)	128		
	No	48 (43.6%)	62 (56.4%)	110		
Perceived effect of smartphone use	Positive	31 (33.7%)	61 (66.3%)	92	χ² = 20.80	<0.001*
	Negative	13 (12.6%)	90 (87.4%)	103		
	Neutral	71 (37.8%)	117 (62.2%)	188		
Creative activity frequency	Occasionally	56 (35.7%)	101 (64.3%)	157	χ² = 9.29	0.010*
	Frequently	25 (36.8%)	43 (63.2%)	68		
	Rarely or never	34 (21.5%)	124 (78.5%)	158		
Desire to reduce smartphone use	Yes	55 (23.7%)	177 (76.3%)	232	χ² = 11.43	0.003*
	No	32 (41.6%)	45 (58.4%)	77		
	Not sure	28 (37.8%)	46 (62.2%)	74		

**Table 4:** Univariate associations and crude odds ratios for risk of smartphone addiction (N = 383) Each one-step improvement in sleep quality or creative engagement is significantly associated with lower odds of smartphone addiction. * Represents a significant p-value.

Variable	Category	OR (95% CI)	p-value
Depression risk (PHQ-2 ≥ 3)	Probable major depression (Vs not likely depressed)	3.83 (2.35–6.25)	<0.001*
Anxiety risk (GAD-2 ≥ 3)	Probable anxiety disorder (Vs likely no anxiety disorder)	3.11 (1.91–5.06)	<0.001*
Physical activity	Physically active (Vs inactive)	0.54 (0.34–0.86)	0.009*
Sleep quality	As ordinal variable	0.78 (0.63–0.96)	0.022*
	Poor (Vs terrible as a reference)	2.12 (0.86–5.26)	0.103
	Fair	1.24 (0.56–2.78)	0.593
	Good	0.79 (0.33–1.91)	0.599
	Excellent	0.64 (0.23–1.80)	0.395
Social Connection	At risk (vs. not at risk)	1.08 (0.69–1.69)	0.737
Emotional Support	At risk (vs. not at risk)	1.28 (0.81–2.00)	0.290
Loneliness/Social Isolation	At risk (vs. not at risk)	2.77 (1.58–4.86)	<0.001*
Academic performance affected	Yes, negatively (vs. positively as reference)	3.72 (1.98–7.00)	<0.001*
	No	1.05 (0.56–1.97)	0.875
Perceived effect of smartphone use	Negative (vs. positive as a reference)	3.50 (1.65–7.41)	0.001*
	Neutral	1.49 (0.75–2.97)	0.259
Creative activity frequency	As ordinal variable	0.66 (0.49–0.89)	0.006*
	Occasionally (vs. rarely/never as a reference)	0.49 (0.30–0.82)	0.006*
	Frequently	0.47 (0.25–0.88)	0.018*
Desire to reduce smartphone use	No (vs. yes as a reference)	0.44 (0.24–0.82)	0.009*
	Not sure	0.56 (0.31–1.01)	0.053
